# New Haven Medical Association

**Published:** 1842-09-24

**Authors:** 


*New Haven Medical Association.* The physicians of New Haven appear to enjoy a harmonious and agreeable intercourse. We learn from the published notice of their association, "that since its foundation, no physician of respectability has ever established himself in the city without becoming a member of the Association; and for a period of thirty-two years, but *two* of its members have *withdrawn* from its connection. The Association numbers at this time eighteen out of the twenty regular physicians in the city. It is confidently believed that, from the manner of organization of the Association, and the way of conducting its meetings, it conduces more to raise the standard of the profession to that grade to which it is entitled, than any similar Association in the country. It is probably owing to this fact, that the physicians of New Haven are on better terms with each other, than in any other city in the Union."

The articles of the constitution of the Association strike us as judicious. The "fee table" we should be glad to see, at least in one or two items, raised to a higher grade. The obstetric fee of five dollars, the gonorrhœa fee of five dollars, the syphilis fee of eight dollars ought to be increased.

